# Development and Validation of a Calibration Gauge for Length Measurement Systems

**DOI:** 10.3390/ma12233960

**Published:** 2019-11-29

**Authors:** Francisco Javier Brosed, Raquel Acero Cacho, Sergio Aguado, Marta Herrer, Juan José Aguilar, Jorge Santolaria Mazo

**Affiliations:** Design and Manufacturing Engineering Department, Universidad de Zaragoza, María Luna 3, 50018 Zaragoza, Spain; racero@unizar.es (R.A.C.); saguadoj@unizar.es (S.A.); 680722@unizar.es (M.H.); jaguilar@unizar.es (J.J.A.); jsmazo@unizar.es (J.S.M.)

**Keywords:** calibration artifact, kinematic support, dimensional metrology, machine tool, length measurement

## Abstract

Due to accuracy requirements, robots and machine-tools need to be periodically verified and calibrated through associated verification systems that sometimes use extensible guidance systems. This work presents the development of a reference artefact to evaluate the performance characteristics of different extensible precision guidance systems applicable to robot and machine tool verification. To this end, we present the design, modeling, manufacture and experimental validation of a reference artefact to evaluate the behavior of these extensible guidance systems. The system should be compatible with customized designed guides, as well as with commercial and existing telescopic guidance systems. Different design proposals are evaluated with finite element analysis, and two final prototypes are experimentally tested assuring that the design performs the expected function. An estimation of the uncertainty of the reference artefact is evaluated with a Monte Carlo simulation.

## 1. Introduction

Volumetric verification is a verification technique to improve the accuracy of machine tools (MTs) and robots based on indirect measurement [1]. It uses the combined effect of all geometric errors through a parameter identification process [2]. Many studies have been carried out for its application to coordinate measurement machines (CMMs) and machine tools (MTs) [3,4]. The increasing implementation of this verification technique in the field of machine tool verification has led to the development of verification procedures that depend on different factors such as the type of machine, the non-geometric errors of the machine, the system and measurement technique applied, etc. [5]. The result of the equipment’s verification is linked to the calibration of the measurement system used, procedure which is normally carried out in accordance with the applicable standards. This applies to measuring instruments commonly used in volumetric verification such as laser trackers [6]. However, in some cases, the lack of guidelines or standards makes it necessary to develop internal calibration procedures and to use specific reference gauges [7,8].

Therefore, this work presents the development of a reference artefact to calibrate extensible guidance systems used in machine tool and robot verification procedures. The reference artefact materializes several working positions and lengths with a fixed reference origin. The reference origin consists of a nest for a precision sphere, and the working positions include different nests with precision spheres and kinematic couplings. The mechanical repeatability of the reference artefact for the nests’ positioning in the different working locations is achieved with kinematic couplings configuration of spheres and cylinders. The design of the artefact will also compensate the errors associated with its deflections [9,10]. In [9], an analysis of the measured length of long artifacts is showed, and the use of tubes instead of solid bars is recommended to reduce the elongation due to self-loading. In [10], the author gives an estimation of the location of the supports to obtain parallel surfaces at the end of a bar (Airy points) and another option (Bessel points) to obtain the minimum change in length.

The paper is structured as follows. Firstly, the authors analyze the requirements of the design and the structure of the reference artefact. Secondly, it is performed an evaluation of the different gauge design proposals by means of a finite element simulation in Solid Edge. In this analysis, the displacement generated in the gauge due to the load application is measured for each case. Then, the design proposals selected are manufactured by 3D printing, and these prototypes are used in the experimental testing and measured with a CMM (coordinate measurement machine). Finally, after optimizing the design with the feedback of simulation and experimental testing, the paper presents an uncertainty estimation of the designed calibration system.

## 2. Materials and Methods

**Figure 1:**
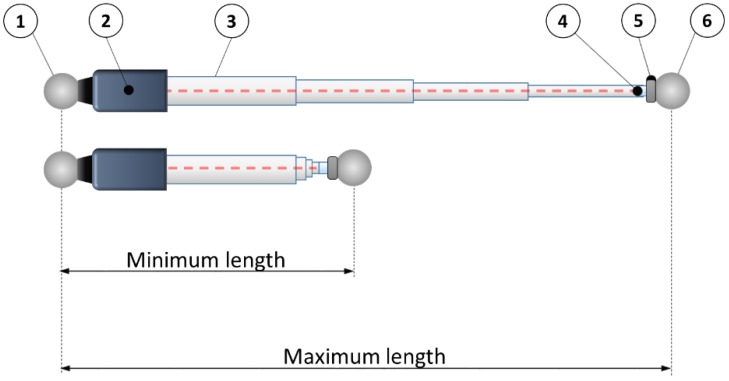
Scheme and components of the measurement instrument. (1) sphere fixed to the instrument; (2) interferometer; (3) telescopic system; (4) retroreflector; (5) magnetic holder; (6) sphere fixed to the machine tool.

The calibration artefact has to materialize the calibration positions for a length measurement instrument. The instrument consists of a system that measures the distance between two spheres. One of the spheres is fixed to the instrument and the other is fixed to the machine tool, robot or coordinate measuring machine under verification. As it can be seen in Figure 1, the gauge is composed of a sphere (1) and a support (5) to hold the sphere fixed to the machine tool under verification (6), being both located at the edges of the artefact. Between both sides, there is an interferometer (2) to measure the different distances that will be materialized in the gauge. These different lengths are achieved with a telescopic system (3) that also assures the alignment of the interferometer and the retroreflector (6). The interferometer is located on the left side of the gauge closed to the fix sphere (1). The retroreflector will be located on the other side of the system close to the sphere fixed to the machine tool. The calibration artefact should be able to calibrate measurement instruments with a measurement range from 400 mm to 1600 mm (max. and min. in Figure 1). Once calibrated, the instrument will give the distance between the centers of the two spheres.

The calibration artefact has a fixed magnetic sphere-holder to lock the position of the sphere fixed to the instrument (1) and several kinematic supports to obtain a repeatable positioning of a sphere. When the sphere of the instrument (1) is locked in the magnetic sphere holder and the other side of the instrument (5) reaches the sphere fixed to the machine tool, a calibrated length materializes in the gauge. The defined nominal lengths of the calibration artefact range from 400 to 1600 mm.

During the calibration of the measurement instrument, the calibration artefact rests in a flat surface. Therefore, the artefact incorporates three support legs on its base to assure its stability in the calibration process.

The main components under analysis in the design of the gauge are the following: the position of the support points to minimize the deformation in the length measurement, the kinematic couplings support that allows the movable sphere positioning with high repeatability and the mechanical structure to materialize the calibration lengths.

**Figure 2:**
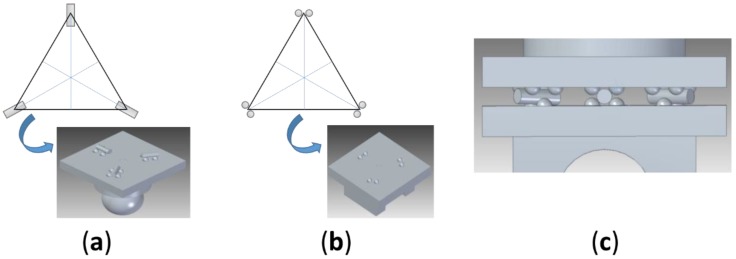
Kinematic support for the mobile sphere. (**a**) Distribution and orientation of the cylinders; (**b**) distribution and orientation of the spheres; (**c**) model of the kinematic support mounted, contact between spheres and cylinders.

During the calibration of the measurement instrument, we need a repeatable positioning of the movable sphere to materialize the calibration positions. For this purpose, a kinematic base has been designed with calibrated spheres and cylinders (6-points 3-cylinders). The kinematic contact has two parts (upper and lower). In the lower part, six spheres are fixed in three pairs located at 120° meanwhile in the upper part, three cylinders are fixed with its axis located at 120° and pointing to the center of the geometrical distribution (Figure 2). Each interface provides two constraints, totaling six constraints for the system. The best stability is achieved when the axes of the contact planes bisect the coupling triangle with each interface as a vertex of this triangle. Four spheres secure the position of the cylinders in the upper part. The upper and lower parts are fixed with magnets located in the center of the geometrical distribution (in the upper and lower part respectively).

The main element of the artefact is a tube that goes through the other parts of the assembly. The junction between the tube and the other parts (magnetic holder or kinematic support for the magnetic holder) is materialized with a flange.

**Figure 3:**
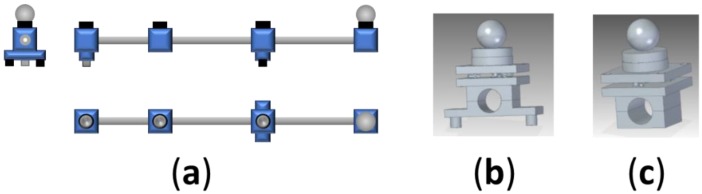
(**a**) Single tube structure for the calibration artefact; (**b**) flange of the single bar artefact with standing legs; (**c**) flange of the single bar artefact without standing legs.

Two design proposals for the artefact structure are evaluated. The first prototype is a single tube structure in which the line of the measurement points is parallel to the bar and is located beyond the structure (Figure 3a). Each flange has been designed to hold the bar and locate the magnetic holder, in one case, and the kinematic support for the magnetic holder, in the rest of the cases, defining each measurement position (Figure 3b,c).

**Figure 4:**
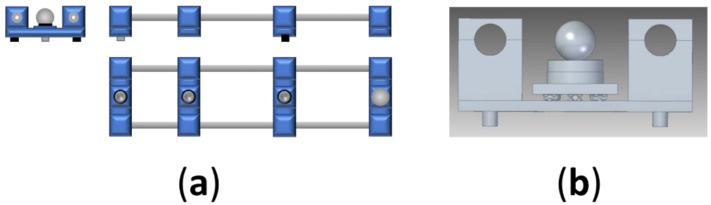
(**a**) Double tube structure for the calibration artefact; (**b**) flange for the double bar structure with the base and he kinematic support.

The second prototype is a double tube structure that locates the line of the measurement points between both bars (Figure 4a). The flanges hold the bars and support a base where the magnetic holder is located in the first point and the kinematic supports in the other cases (Figure 4b).

A horizontal bar of great length requires two points of support in the direction of its length to be stable. The position of these standing legs determines the action of the gravity of this bar; depending on how it is supported, measurement errors can be caused [11]. Therefore, if the supports are positioned at the ends, it will warp in the center causing the ends to come closer and tilt upwards. On the contrary, if the two supports are positioned in the middle, the bar will be bent at the ends [9]. From [9] the use of tubes instead of bars to reduce the elongation due to self-loading is taken as well as the location of the spheres in the neutral bending surface.

**Figure 5:**
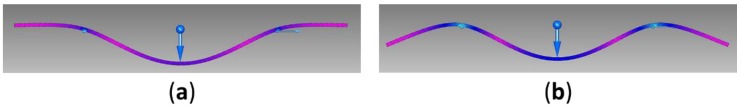
Results of the simulation of a simple bar (aluminum) with two supports following each two methods. (**a**) Simple bar supported at the Airy points; (**b**) simple bar supported at the Bessel points.

The distances between the supports of the bar have been defined using Airy and Bessel methodologies and comparing the results of the deformation. A bar supported at its Airy points has parallel ends and supported at its Bessel points has maximum length due to deflection reduction. The value of the Airy and Bessel points has been taken from [10]. The distance between supports (a) and the position of each support (Lmin and Lmid), for a simple bar of 1600 mm length (L), appears in Table 1, and the deformation obtained in the bar appears in Figure 5.

**Figure 6:**
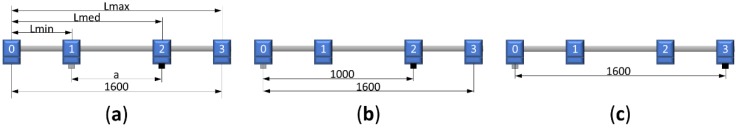
Scheme of the four locations of the supports and the location of the loads in each simulation; there is a load of 1N in point 0 for every simulation and another load of 1N in point 1 for the simulation of Lmin, in point 2 for the simulation of Lmid, and in point 3 for the simulation of Lmax. (**a**) Airy and Bessel points; (**b**) supports located in point 0 (reference) and in Lmid; (**c**) supports located in point 0 (reference) and in Lmax.

Four different positions of the supports are proposed; two of them following the Airy and Bessel methodologies (Figure 6a). The other two configurations locate the supports in the reference flange (point 0, Figure 6b) and in the flange that materializes Lmid (point 2, Figure 6b) in the third case and in the reference flange (point 0, Figure 6c) and in the flange that materializes Lmax (point 3, Figure 6c) in the fourth case.

## 3. Results

This section provides the description of the main results of the components analysis and the development of the artefact. As the previous section indicates, the main components under analysis are the following: the position of the support points to minimize the deformation in the length measurement, the kinematic couplings support that will allow the movable sphere positioning with high repeatability, and the mechanical structure to materialize the calibration lengths.

### 3.1. Design Selection

In order to define the position of the standing legs, a finite element analysis of the deformation of the structure has been carried out (twenty-four simulations, twelve for each prototype, were performed). The study analyses four different positions of the supports, and the deformation occurred when the measurement system was placed in the three different measurement positions (Lmin, Lmid, and Lmax). The measurement system will rest in the reference position and in the position under verification (Lmin, Lmid, or Lmax). Therefore, in the analysis there is a load of 1N in the reference position (position of the magnetic holder, point 0, Figure 6) and another load of the same value in the measurement position for each case. The four positions of the supports are the Airy point (a = 923.76 mm), the Bessel points (a = 895.04 mm, Figure 6a), the supports located in the reference position and in the Lmid (Figure 6b), and finally, the supports located in the reference position and in the Lmax (Figure 6c). All the combinations make twenty-four simulations (2 prototypes, 4 supporting leg configuration and 3 load configurations).

The material properties taken into account for the structural analysis are shown in Table 2.

The increment of the measurement distance or the measurement error Δ*L_MEAS_* in Equation (1) characterizes the deformation of the structure.

(1) $$\Delta L_{MEAS}=\sqrt{{\left(L_{n}+\Delta x_{n}-\Delta x_{0}\right)}^{2}+{\left(\Delta y_{n}-\Delta y_{0}\right)}^{2}+{\left(\Delta z_{n}-{\Delta z}_{0}\right)}^{2}},$$

where *L_n_* is the nominal distance of the measurement point (*n* = Lmin, Lmid and Lmax); (Δ*x_n_*, Δ*y_n_*, Δ*z_n_*) are the displacements of the measurement point due to the deformation of the structure, and (Δ*x_0_*, Δ*y_0_*, Δ*z_0_*) are the displacements of the reference point due to the deformation of the structure.

**Figure 7:**
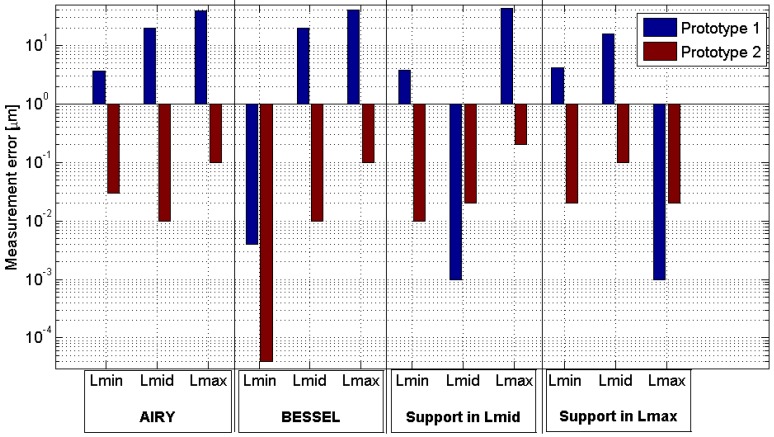
Measurement error in µm for the twelve cases for each two prototypes. These results have been obtained after the analysis of the prototypes using finite elements software.

Combining the four proposed positions of the supports and the three different pairs of loads, twelve values of simulated measurement error have been obtained for each prototype after the analysis of the prototypes using finite elements software (Solid Edge ST8, Siemens PLM Software, Plano, TX, USA) (Figure 7).

The localization of the spheres beyond the structure line amplifies the measurement error due to the deformation in prototype 1. Based on that, prototype 1 was discarded and the results shown in the paper correspond to prototype 2. The measurement errors in prototype 2 are minimum using the Bessel points and do not exceed from 0.1 µm, value obtained when the system is loaded in Lmax position (points 0 and 3, Figure 6) and lower in the other cases (Lmin and Lmid) (according with the simulation results using finite elements software (Solid Edge ST8, Siemens PLM Software, Plano, TX, USA)).

**Figure 8:**
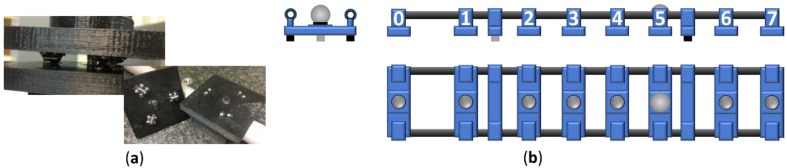
(**a**) Kinematic supports samples manufactured by additive manufacturing; (**b**) prototype 3.

After the simulation with the 3D models of prototypes 1 and 2, a kinematic support prototype was manufactured by additive manufacturing (Figure 8) and tested using a CMM. The kinematic supports have been tested measuring the repeatability of the manufactured kinematic supports with the spheres and the cylinders. The position measurement repeatability of each location obtained after ten iterations was 8 µm (measured with a CMM).

Once the kinematic supports have been tested (repeatability measured with a CMM), and the adequacy of the Bessel points for this application has been proved (simulation using finite elements software (Solid Edge ST8, Siemens PLM Software, Plano, TX, USA), we manufactured a new design with aluminum flanges and carbon fiber structure tubes (27′’ diameter, 1830 mm length). The number of measurement points increments to seven from three in the previous prototypes (Table 3). In this case, the values of the measurement errors obtained for each measurement position are under 0.1 µm (measured with CMM).

### 3.2. Manufacture, Assembly, and Performance

The flanges, the bases where the kinematic supports are located, and the standing legs of the artefact were made of Aluminum 6061 for the prototype 3. The flanges join the carbon fiber tubes with the base that contains the magnetic holder for the position A (n = 0, Figure 8b) and with the base that contains the kinematic supports for the rest of the cases, positions B to H (n from 1 to 7, Figure 8b).

**Figure 9:**
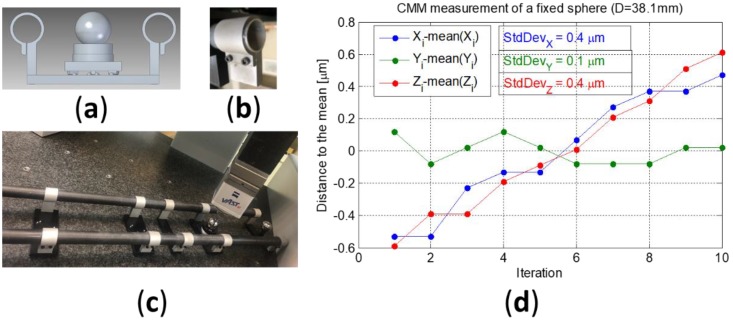
(**a**) Model of the flanges in prototype 3; (**b**) detail of the flange embracing a tube; (**c**) prototype 3 with the new flanges and carbon fiber tubes during performance test in coordinate measurement machine (CMM); (**d**) CMM measuring a fixed sphere (Diameter 38.1 mm), distance to the mean of the ten iterations in mm. Standard deviation values of each coordinate are included next to the legend.

The flange geometry has been redesigned to adequate it to a wire EDM manufacturing process (Figure 9a,b).

The position of the spheres has been measured with a coordinate measurement machine (CMM) to obtain the uncertainty of the calibration artefact. First, the repeatability of the CMM measuring a sphere with a diameter of 38.1 mm (1½’’) has been estimated in 0.4 µm. The measurement position number 3 (Figure 8b) was measured ten times with the CMM without removing the sphere from the kinematic support (Figure 9d).

**Figure 10:**
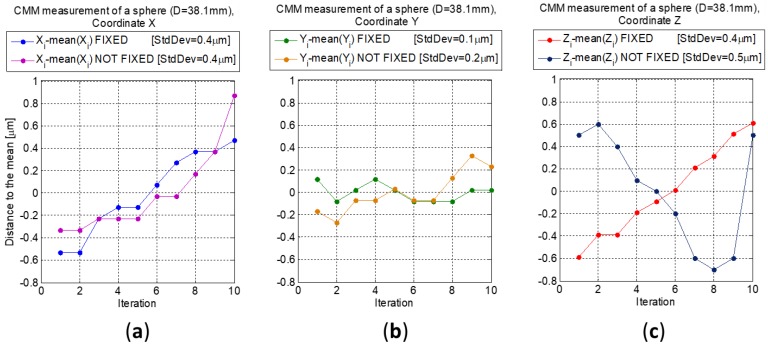
CMM measuring a sphere (Diameter 38.1 mm), distance to the mean of the ten iterations in mm in each coordinate. Standard deviation values of each coordinate (fixed and not fixed) are included in the legend. The results are compared with those obtained measuring a fixed sphere. (**a**) Distance to the mean of the ten iterations in mm in X coordinate; (**b**) Distance to the mean of the ten iterations in mm in Y coordinate; (**c**) Distance to the mean of the ten iterations in mm in Z coordinate.

A second measurement with ten iterations was carried out assembling and disassembling the kinematic support with the sphere located in position number 3 (not fixed sphere, Figure 10). The results are compared with those obtained without removing the kinematic support (fixed sphere, Figure 10).

The standard deviation values of the sample in X, Y, and Z coordinates are 0.4, 0.1, and 0.4 µm, respectively, without removing the kinematic support (fixed sphere). Disassembling the kinematic support with the sphere located in position number 3 (not fixed sphere) are 0.4, 0.2, and 0.5 µm.

Z coordinate is more sensible to the movements when mounting and demounting the kinematic support but, in any case, the standard deviation of the sample is low enough for the application.

**Figure 11:**
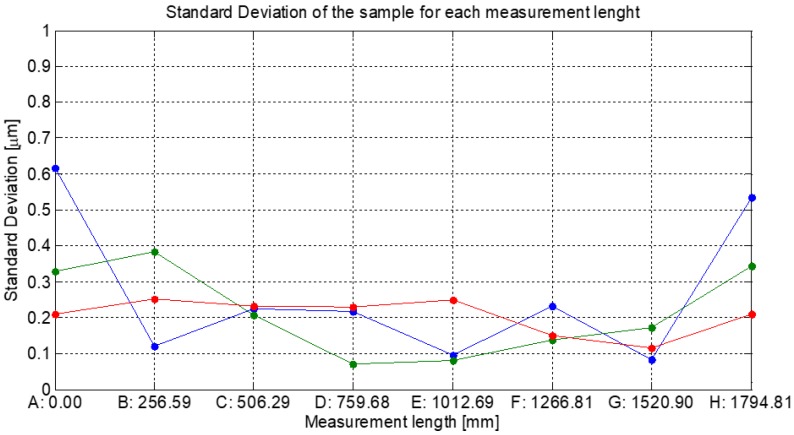
CMM measuring a sphere (Diameter 38.1 mm) in each position of the artefact. The graph shows the standard deviation of the sample (ten iterations) for each measurement length (X, Y, and Z coordinates). The abscissa identifies the mean value of the measurement length of each position (from A to H).

The next step, after measuring the repeatability of the kinematic supports, is to check the effect of the deformation in the measurement length corresponding to each sphere position. To evaluate this effect, the measurement of the reference position (A, number 0 in Figure 8b) and the other positions has been carried out moving the sphere with the kinematic support from B (position number 1 in Figure 8b) to H (position number 7 in Figure 8b). The procedure is repeated ten times. The measurement results allow estimating the repeatability of the measurement length in each position as the standard deviation of ten repetitions in each position (Figure 11).

### 3.3. Calibration Artefact Uncertainty Estimation with Monte Carlo Simulation

Monte Carlo (MC) method is widely used in measurement uncertainty estimation procedures [12,13], for example applied to coordinate measuring machines (CMMs) uncertainty analysis [14], [15,16,17]. In this work, we used the MC simulation to estimate the uncertainty of the reference artefact in the calibration of length measurement systems. The uncertainty values have been calculated according with the Guide to the expression of uncertainty in measurement GUM [18,19] using a confidence level of 95% (k = 2).

The input data for the MC simulation method are the probability distributions of the variability of the different error sources. In this case, the main error source is the variability of the positioning of each calibration point.

**Figure 12:**
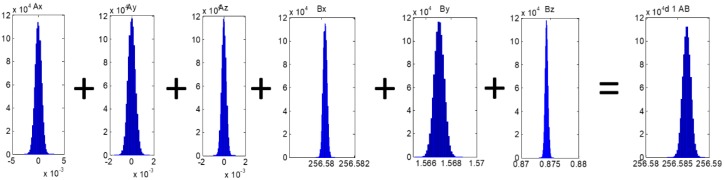
Distribution of the values for each set of coordinates of A and B positions. The distance between this two positions materializes the measurement length number 1 (d1, AB). The distribution of the measurement length can be obtained by simulation with the Monte Carlo method.

The nominal value of each position coordinate is the mean value obtained from the CMM measurement. The standard deviation of the distribution of each position is also the standard deviation of the CMM measurements for each position (Figure 11). Then, each position (from A to H) is measured with a CMM and the repeatability of the X, Y, and Z coordinates is evaluated modelling its distribution as a normal distribution. When the effect of the distribution of each variable that influences the measurement length materialized by the calibration artefact is considered, the distribution of the measurement result can be estimated (Figure 12).

**Figure 13:**
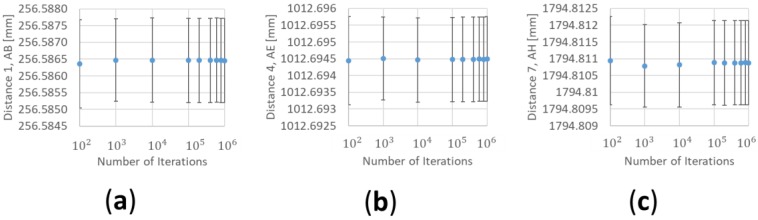
Evolution of the results as the number of iterations increase from 10^2^ to 10^6^. (**a**) Results for the first measurement length materialized between point A and point B of the calibration artefact; (**b**) results for the fourth measurement length materialized between point A and point E of the calibration artefact; (**c**) results for the seventh measurement length materialized between point A and point H of the calibration artefact.

The results of the Monte Carlo simulation also depend on the number of iterations carried out. When the number of iterations is low, the results are not representative but as the number of results increases, the values obtained for the uncertainty converge (Figure 13).

**Figure 14:**
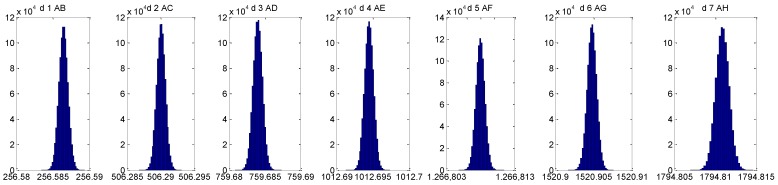
The uncertainty distribution for each measurement length (from distance d1, AB to distance d7, AH).

The results of the MC simulation are shown in Table 4 including the uncertainty value for each length measurement. The uncertainty distribution for each measurement length (from distance between n=0 to n=1 in Figure 8b, distance 1 AB, to distance between n=0 to n=7 in Figure 8b, distance 1 AH) is shown in Figure 14 obtaining values below ±1.60 µm for all the positions in the study.

## 4. Discussion

This work presented the design, development, manufacturing, and experimental validation of a reference artefact to calibrate extensible guidance systems used in machine tool and robot verification procedures. The artefact uses spheres and spherical nests with kinematic supports that assure the high repeatability of the system. Different design proposals were evaluated with finite element analysis, and two final prototypes were experimentally tested assuring that the design of kinematic couplings performs the expected function. The paper finally presents the uncertainty estimation of the calibration artifact using a Monte Carlo simulation (MC).

We could conclude from the results of the Monte Carlo simulation that the calibration uncertainty of the artefact designed for length measurement systems could be adequate for the application, considering tests carried out in a horizontal position.

The calibration artefact presented in this work can be used to test the telescopic system not only in a horizontal position but also by varying the angle and reaching an upright position. Therefore, simulation and experimental validation would be necessary in these conditions in the future, although it is expected that the configuration of the most precise calibration artefact would be the same as the one presented in this paper for horizontal tests.

## Figures and Tables

**Table 1:** Values of the Airy and Bessel point.

Parameter	Airy	Bessel
L (mm)	1600	1600
Factor	0.57735	0.55940
a (mm)	923.76	895.04
Lmin (mm)	338.12	353.5
Lmid (mm)	1261.88	1247.88

**Table 2:** Material properties for prototypes 1 and 2 (aluminum 6061) and prototype 3 (carbon fiber).

Property	Al 6061	Carbon Fiber
Density (T/m^3^)	2.7	1.6
Young Module (GPa)	68.9	393.3
Poisson Coeficient	0.330	0.100

**Table 3:** Bessel and measurement points for prototype 3 (proposed nominal values).

A: *n* = 0	B: *n* = 1	Bessel 1	C: *n* = 2	D: *n* = 3	E: *n* = 4	F: *n* = 5	Bessel 2	G: *n* = 6	H: *n* = 7
0.00	280.00	396.54	535.00	785.00	1040.00	1295.00	1403.46	1545.00	1800.00
(mm)									

**Table 4:** Bessel and measurement points for prototype 3 (proposed nominal values).

Position	Number, n	Measurement length (mm)	Uncertainty (k = 2) (µm)
B	1	256.586	±1.25
C	2	506.29	±1.30
D	3	759.684	±1.30
E	4	1012.694	±1.20
F	5	1266.808	±1.30
G	6	1520.905	±1.20
H	7	1794.811	±1.60
